# Evolution of ocular defects in infant macaques following in utero Zika virus infection

**DOI:** 10.1172/jci.insight.143947

**Published:** 2020-12-17

**Authors:** Glenn Yiu, Sara M. Thomasy, M. Isabel Casanova, Alexander Rusakevich, Rebekah I. Keesler, Jennifer Watanabe, Jodie Usachenko, Anil Singapuri, Erin E. Ball, Eliza Bliss-Moreau, Wendi Guo, Helen Webster, Tulika Singh, Sallie Permar, Amir Ardeshir, Lark L. Coffey, Koen K.A. Van Rompay

**Affiliations:** 1 Department of Ophthalmology & Vision Science, School of Medicine, and; 2 Department of Surgical and Radiological Sciences, School of Veterinary Medicine, University of California, Davis, Davis, California, USA.; 3 California National Primate Research Center, Davis, California, USA.; 4 Department of Pathology, Microbiology and Immunology, School of Veterinary Medicine, and; 5 Department of Psychology, University of California, Davis, Davis, California, USA.; 6 Duke Human Vaccine Institute, Duke University Medical Center, Durham, North Carolina, USA.

**Keywords:** Immunology, Ophthalmology, Neurodevelopment, Retinopathy

## Abstract

Congenital Zika syndrome (CZS) is associated with microcephaly and various neurological, musculoskeletal, and ocular abnormalities, but the long-term pathogenesis and postnatal progression of ocular defects in infants are not well characterized. Rhesus macaques are superior to rodents as models of CZS because they are natural hosts of the virus and share similar immune and ocular characteristics, including blood–retinal barrier characteristics and the unique presence of a macula. Using a previously described model of CZS, we infected pregnant rhesus macaques with Zika virus (ZIKV) during the late first trimester and characterized postnatal ocular development and evolution of ocular defects in 2 infant macaques over 2 years. We found that one of them exhibited colobomatous chorioretinal atrophic lesions with macular and vascular dragging as well as retinal thinning caused by loss of retinal ganglion neuron and photoreceptor layers. Despite these congenital ocular malformations, axial elongation and retinal development in these infants progressed at normal rates compared with healthy animals. The ZIKV-exposed infants displayed a rapid loss of ZIKV-specific antibodies, suggesting the absence of viral replication after birth, and did not show any behavioral or neurological defects postnatally. Our findings suggest that ZIKV infection during early pregnancy can impact fetal retinal development and cause congenital ocular anomalies but does not appear to affect postnatal ocular growth.

## Introduction

Zika virus (ZIKV) is a mosquito-transmitted flavivirus that was first isolated from a rhesus macaque in the Zika Forest of Uganda in 1947. ZIKV received worldwide recognition when a surge of congenital birth defects occurred closely after a ZIKV outbreak in Brazil in 2015. The rapid expansion of the outbreak in the Americas led to its declaration by the World Health Organization as a public health emergency in 2016 (1). The spectrum of fetal and neonatal anomalies, including microcephaly, ocular defects, musculoskeletal contractures, and neurologic deficits, combined with a diagnosis of prenatal ZIKV infection, together constitute congenital Zika syndrome (CZS). The predilection for CNS abnormalities in CZS is explained by the tropism of ZIKV for neural progenitor cells (2). Studies indicate this may be due to the binding of the viral RNA genome to the RNA-binding protein Musashi-1 that is involved in neurodevelopment and is highly expressed in these precursor neurons (3). Multiple strategies enable ZIKV to evade host innate immune responses to allow spread to the placenta of the mother, and to traverse both the blood–cerebrospinal fluid barrier in the choroid plexus and blood–brain barrier of the fetus (4). Because many infants whose mothers are ZIKV-infected during pregnancy are born without microcephaly or detectable viral RNA in fluids, but may develop neurologic problems later in life, long-term monitoring of these young children is essential (5–7).

A unique feature of CZS is the high frequency of ocular malformations, particularly in patients with microcephaly (8–17). Ocular findings in these infants primarily impact posterior segment structures such as the retina, choroid, and optic nerve, including chorioretinal atrophy, torpedo maculopathy, retinal vessel tortuosity, peripapillary atrophy, and optic disc hypoplasia (8, 9, 15, 18–20). Central retinal thinning has also been observed on in vivo imaging using spectral domain–optical coherence tomography (SD-OCT), particularly in the ganglion cell layer (GCL) that consists of axonal projections from the eye to the brain (21). Other eye findings include anterior segment abnormalities, such as iris coloboma, lens subluxation, cataract, and glaucoma, as well as neuro-ophthalmic deficits, such as oculomotor dysfunction and loss of pupillary response (9, 22–24). Although acquired ZIKV infection can cause intraocular inflammation such as conjunctivitis, iridocyclitis, and posterior uveitis (13, 25, 26), these findings have not been reported in congenital cases. Maternal symptoms of ZIKV during the first trimester of pregnancy are associated with a higher frequency of ocular abnormalities, which, similar to other birth defects in CZS, may result from the significant neural cell proliferation and differentiation occurring during this critical period (8, 18). ZIKV can bypass the developing blood–retinal barrier by infecting neural progenitor cells, and it can also infect other cell types located in the inner and outer blood–retinal barrier, including retinal vascular endothelial cells, pericytes, Muller glia, and retinal pigment epithelium (RPE) (27, 28).

Because rodents are not reservoir hosts for ZIKV, modeling CZS is limited by the inability of ZIKV to replicate efficiently in pregnant outbred mice, often requiring the use of immunodeficient mice. Moreover, ocular anatomy and development in mice differ significantly from those of humans, particularly due to the absence of a cone-rich macula that enables high-acuity daytime vision, which uniquely exists in primates (29). ZIKV infection of pregnant rhesus macaques is a highly relevant animal model of CZS because it recapitulates many features of human ZIKV infection and CZS, including time course of viremia, maternal neutralizing antibody responses, rates of vertical transmission, and development of placental and fetal neurologic abnormalities and fetal loss, although microcephaly has not been observed in these animals (30–38). This model has also been used successfully to demonstrate the efficacy of antiviral interventions, such as active and passive immunization strategies, in reducing transplacental transmission and the harmful effects of in utero infection (39, 40).

Prior reports of ocular findings in fetal macaques born to ZIKV-infected pregnant animals included choroidal colobomas, retinal dysplasia, and possible anterior segment dysgenesis. However, these features were described based only on postmortem histology from fetuses that either underwent fetal demise due to preterm premature rupture of membranes on gestational day (GD) 95 (41) or collected at the end of gestation (34, 35). To our knowledge, no studies have studied CZS-related ocular pathology in postnatal infant macaques, employed live imaging, or monitored the progression of such infants over prolonged periods of time after birth. In this study, we used a previously developed pregnancy macaque model of CZS that was designed to reliably induce fetal infection at defined times, by inoculating the pregnant macaques by both the i.v. and intra-amniotic (IA) routes (31), and describe the evolution of ocular findings in 2 infant macaques over 2 years after birth.

## Results

### ZIKV infection of pregnant macaques and prolonged ZIKV presence in amniotic fluid.

**Figure 1:**
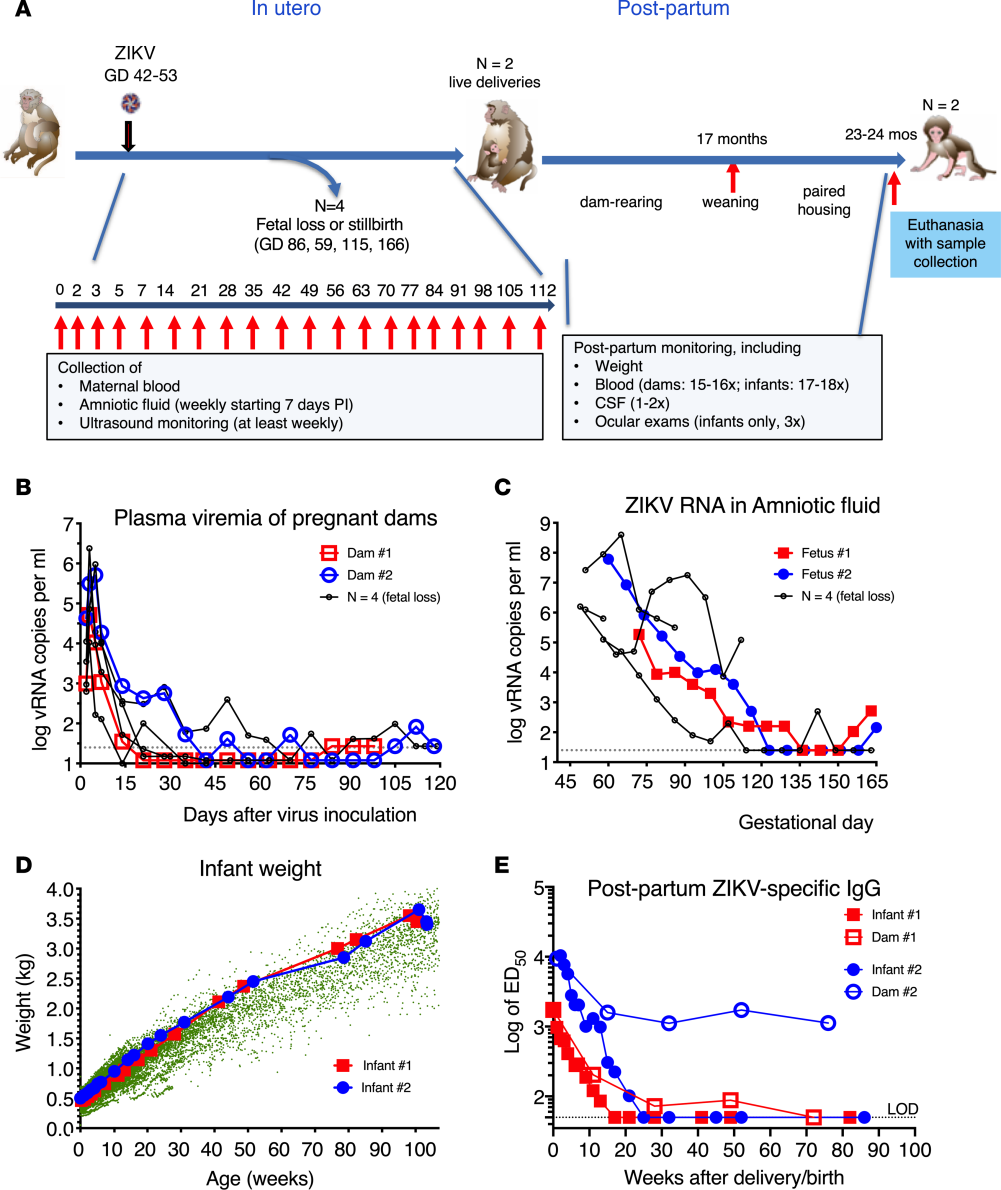
History of infant macaques exposed in utero to ZIKV infection. (**A**) Schematic of experimental design. Pregnant macaques were inoculated by both i.v. and intra-amniotic routes between GDs 42 and 53 followed by frequent monitoring. Whereas 4 dams had fetal loss or stillbirth, the other 2 animals delivered infants that were dam-reared, subsequently weaned and then housed together until they were euthanized at approximately 2 years of age. The patterns of viral RNA levels in plasma (**B**) and amniotic fluid (**C**) of the pregnant dams that delivered live infants were similar to those for animals whose fetuses died and reflects prolonged virus replication. The dotted lines show the limit of detection. (**D**) The 2 ZIKV-exposed infants had normal weight gain. Green dots indicate historical control data (15,585 data points collected from *n* = 284 female animals over the first 2 years of life). (**E**) Anti-ZIKV antibodies in plasma of dams and infants measured by whole-virion ELISA, showing rapid loss of ZIKV IgG in congenitally exposed infants after birth and gradual decline of IgG in ZIKV-infected dams. Magnitude of ZIKV-specific IgG is expressed as the log of ED_50_. ZIKV, Zika virus; GDs, gestational days; ED_50_, 50% of maximal effective dilution.

The 2 infants described in this manuscript were part of a study in which 6 pregnant macaques were each inoculated once, between GDs 42 and 53 (corresponding to the first trimester of pregnancy) with 2000 PFUs of 2 ZIKV strains isolated from 2015 outbreaks, by both the i.v. and IA routes (Figure 1A). Of the 6 animals, 4 animals (inoculated between GDs 42 and 51) experienced early fetal loss (*n* = 3) or stillbirth (*n* = 1), the findings of which have been previously described (30). By contrast, the 2 other pregnant dams (dam no. 1 and dam no. 2, inoculated on GD 51 or GD 53, respectively) had no clinical symptoms and each gave birth to a female infant (infant no. 1 and no. 2, respectively) by natural delivery on GDs 168 and 171, respectively. Both these dams had patterns of high-peak plasma viremia at 5 or 6 log_10_ vRNA copies per mL and prolonged detection of viral RNA in amniotic fluid samples that decreased toward the end of pregnancy in a pattern similar to the 4 dams that lost their fetus or infants (Figure 1, B and C) and to historical data of animals inoculated by these same routes (31). The 2 fetuses that survived showed normal fetal growth and no evidence of microcephaly, as determined by frequent ultrasound monitoring of biparietal diameter (values were within or above the mean ± 2 SD range of uninfected fetuses; data not shown).

### Postnatal course and ZIKV-specific antibody detection in exposed infant macaques.

Upon delivery, both infants looked visibly normal, had normal birth weight (460–500 g, infant no. 1 and infant no. 2, respectively) relative to newborn macaques born at the same facility, and were dam reared. They were housed with their mothers until approximately 17 months of age and then pair housed together until time of euthanasia at approximately 2 years of age. Throughout that time, both animals had normal weight gain (Figure 1D). Both juvenile macaques were tested on a panel of behavioral tests to index differences in affective reactivity and cognition and showed no obvious abnormal behavior compared with 2 age-matched, dam-reared, and weaned juvenile macaques (Bliss-Moreau et al., unpublished observations).

Blood samples were collected regularly (≥15 time points) from the 2 dams and their 2 infants between 2 days after delivery until euthanasia. None of the maternal and infant plasma samples had detectable ZIKV RNA. In addition, several CSF and urine samples and spleen and lymph node specimens collected from both infants at the time of necropsy were tested, and none had detectable viral RNA.

ZIKV-specific IgG antibodies were measured via whole virion ELISA. Concentration of anti-ZIKV IgG in the neonatal macaques shortly after delivery were similar to titers in their mothers (titers of 1:1649 to 1:10,389), suggesting passive transplacental transfer of maternal IgG into the infants’ circulation (Figure 1E). ZIKV-specific IgG in infants then declined with a half-life of approximately 1.9 weeks, which is similar to the half-life previously described for passively acquired rhesus macaque antibodies in infant macaques (42), and became undetectable (titer less than 1:50) by 6 months of age. Although we cannot exclude that the infants may have made their own antibody response in utero, which may have been masked by high titers of maternal antibodies at birth, the observation that the infants did not show persistent anti-ZIKV antibodies in plasma after birth suggests that there was no virus replication in these infants after birth, since persistent infection would have led to an increase in ZIKV-specific IgG antibody titers.

Maternal IgG concentrations were higher and persistent as expected. However, for one of the dams (no. 1), antibody levels became undetectable by 72 weeks after delivery, suggesting limited durability of B cell memory responses after early containment of viremia (Figure 1E).

### Ocular biometry in ZIKV-exposed infant macaques.

**Figure 2:**
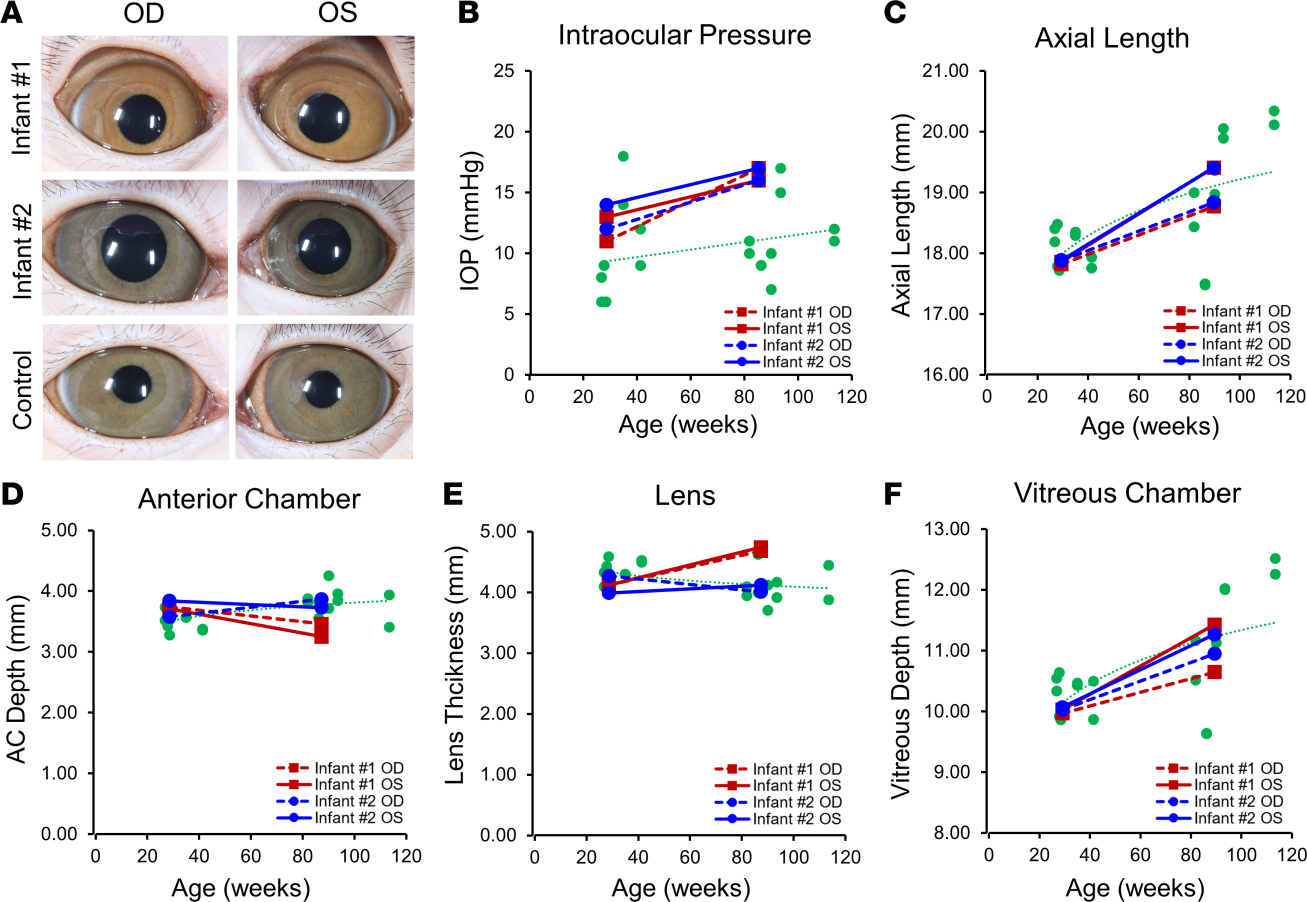
Anterior segment and ocular parameters of ZIKV-infected infant macaques. (**A**) Representative external photographs of anterior segment of right (OD) and left (OS) eyes of 2 infant macaques (no. 1 and no. 2) exposed to ZIKV infection in utero. (**B**) IOPs measured from right (OD, dashed lines) and left (OS, solid lines) eyes from infant no. 1 (red) and no. 2 (blue), compared with eyes from healthy animals (green) and their trendline across similar ages. (**C–F**) Ocular biometric measurements including axial length (**C**), AC depth (**D**), lens thickness (**E**), and vitreous chamber depth (**F**) taken from right (OD, dashed lines) and left (OS, solid lines) eyes from infant no. 1 (red) and no. 2 (blue), compared with eyes from healthy animals (green) and their trendline across similar ages. ZIKV, Zika virus; OD, right eye; OS, left eye; IOPs, intraocular pressures; AC, anterior chamber.

Serial ophthalmic examination of the 2 congenitally ZIKV-exposed infants showed no overt evidence of anterior segment abnormalities on slit lamp biomicroscopy (Figure 2A). Intraocular pressures (IOPs) remained within normal range throughout the study but were slightly above average when compared with normal, age-matched control animals (Figure 2B), although IOP values varied between individuals. Both infants showed normal rates of axial elongation compared with control eyes and published data (43), based on axial lengths measured from ultrasound A-scans at 28 and 82 weeks of age (Figure 2C). However, both eyes of infant no. 1 showed a slight reduction in anterior chamber depth (–0.28 mm and –0.46 mm) and an increase in lens thickness (+0.56 mm and +0.62 mm) between their first and second year of life, based on A-scan biometry, in contrast to the other ZIKV-infected infant (no. 2) and control eyes (Figure 2, D and E), which showed the opposite trend but varied between individual animals. The vitreous chamber elongated in both infants, similar to healthy eyes (Figure 2F). Thus, although the overall axial growth of the ZIKV-infected infant eyes appeared normal, 1 of the 2 animals demonstrated anterior chamber shallowing and lens thickening that did not follow normal postnatal ocular development.

### Chorioretinal lesions in a ZIKV-exposed infant macaque.

**Figure 3:**
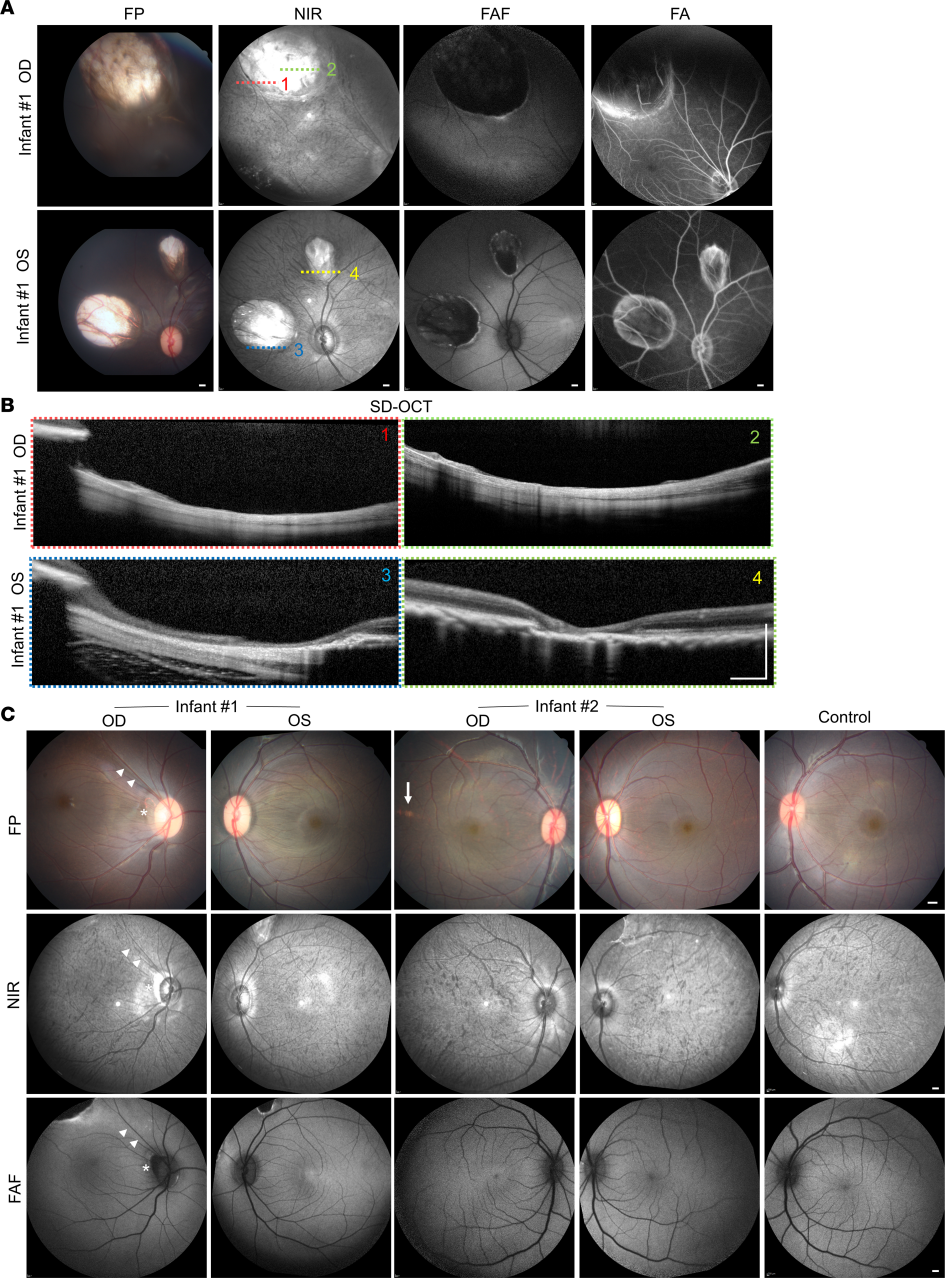
Multimodal imaging of chorioretinal lesions in ZIKV-infected infant macaques. (**A**) Color FP, NIR, FAF, and FA imaging of chorioretinal atrophic lesions in the right (OD) and left (OS) eyes of ZIKV-infected infant no. 1. (**B**) SD-OCT B-scan images of the chorioretinal lesions corresponding to the numbered location (1–4) noted in the NIR images in **A**, taken from the right (OD) and left (OS) eyes of ZIKV-infected infant no. 1. (**C**) FP, NIR, and FAF images of the right (OD) and left (OS) eyes of 2 infant macaques (no. 1 and no. 2) exposed to ZIKV infection in utero. In the top left panel of **C**, the right eye of infant no. 1 showed peripapillary atrophy (asterisk) and superotemporal dragging of the macula and superior vascular arcade (arrowheads) in the direction of the large choreoretinal atrophic lesion seen in top left panel of **A**. The right eye of infant no. 2 showed a small yellowish spot (arrow) that did not appear on NIR or FAF imaging. All scale bars: 500 μm. ZIKV, Zika virus; FP, fundus photograph; NIR, near-infrared; FAF, fundus autofluorescence; FA, fluorescein angiography; OD, right eye; OS, left eye; SD-OCT, spectral domain–optical coherence tomography.

Fundic examination of infant no. 1 by indirect biomicroscopy demonstrated a large, colobomatous chorioretinal atrophic lesion in the superotemporal mid-periphery of the right eye and 2 similar but smaller areas of chorioretinal atrophy nasal and superior to the optic disc of the left eye (Figure 3A). Multimodal imaging demonstrated a lack of choroidal vascular pattern on near infrared (NIR) imaging and absence of RPE-derived fundus autofluorescence (FAF) within these lesions. At the same time, fluorescein angiography (FA) showed staining of the lesion borders without dye leakage, indicating the absence of any neovascular or exudative features (Figure 3A). Live cross-sectional imaging of these lesions using SD-OCT revealed near-complete atrophy of retinal and choroidal layers, with some thin, residual retinal tissues in areas of retinal vessels overlying the scleral wall resembling the typical intercalary membrane seen in chorioretinal colobomas (Figure 3B). Posterior pole examination of infant no. 1 also revealed a crescent-shaped peripapillary atrophy in the right eye, along the same meridian as the large chorioretinal lesion, with superotemporal dragging of the macula and superior retinal vascular arcade (Figure 3C, top left). The macular region of the left eye of infant no. 1 and both eyes of infant no. 2 appeared similar to healthy eyes, with the exception of a small, yellowish spot in the temporal macula of the right eye of infant no. 2, which was not seen on NIR or FAF imaging, suggesting that the spot did not affect the choroid or RPE and is likely nonspecific.

**Figure 4:**
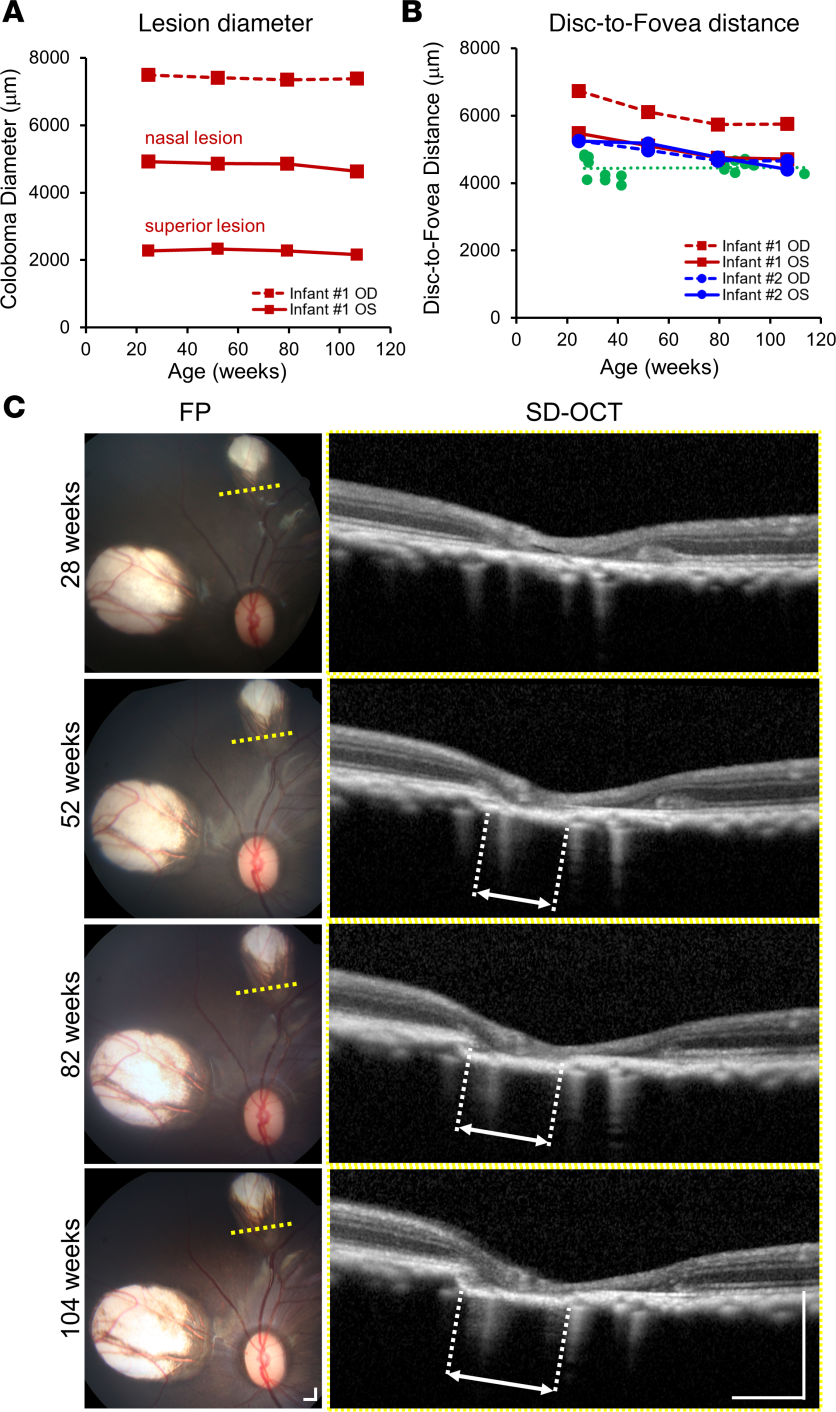
Evolution of chorioretinal lesions and macular dragging in a ZIKV-infected infant macaque. (**A**) Horizontal diameter of chorioretinal lesions and (**B**) disc-to-fovea distances taken from right (OD, dashed lines) and left (OS, solid lines) eyes from 2 ZIKV-infected infant macaques no. 1 (red) and no. 2 (blue), compared with eyes from healthy animals (green) and their trendline across similar ages. (**C**) Serial FP and SD-OCT B-scans corresponding to the yellow dashed lines on the FP taken from the left eye of infant no. 1 at 28, 52, 82, and 104 weeks of age, showing progressive atrophy of outer retinal and choroidal layers as designated by the white dashed lines and double arrows. Scale bar: 500 μm. ZIKV, Zika virus; OD, right eye; OS, left eye; FP, fundus photograph; SD-OCT, spectral domain–optical coherence tomography.

Serial measurements of the 3 chorioretinal atrophy lesions in infant no. 1 showed no noticeable change in lesion diameter during the study period (Figure 4A). However, the disc-to-fovea distance was noticeably longer in the right eye compared with the left eye, eyes of infant no. 2, or control eyes (Figure 4B), likely as a result of the superotemporal dragging of the macula related to the presence of the large chorioretinal atrophy in the superotemporal mid-peripheral retina. Serial SD-OCT imaging of the superior chorioretinal atrophy lesion in the left eye of infant no. 1 demonstrated slow, progressive loss of the outer retinal and choroidal layers at the lesion edge over 2 years (Figure 4C), despite no apparent change in lesion diameter with en face view.

### Retinal thinning in ZIKV-exposed infant macaques.

**Figure 5:**
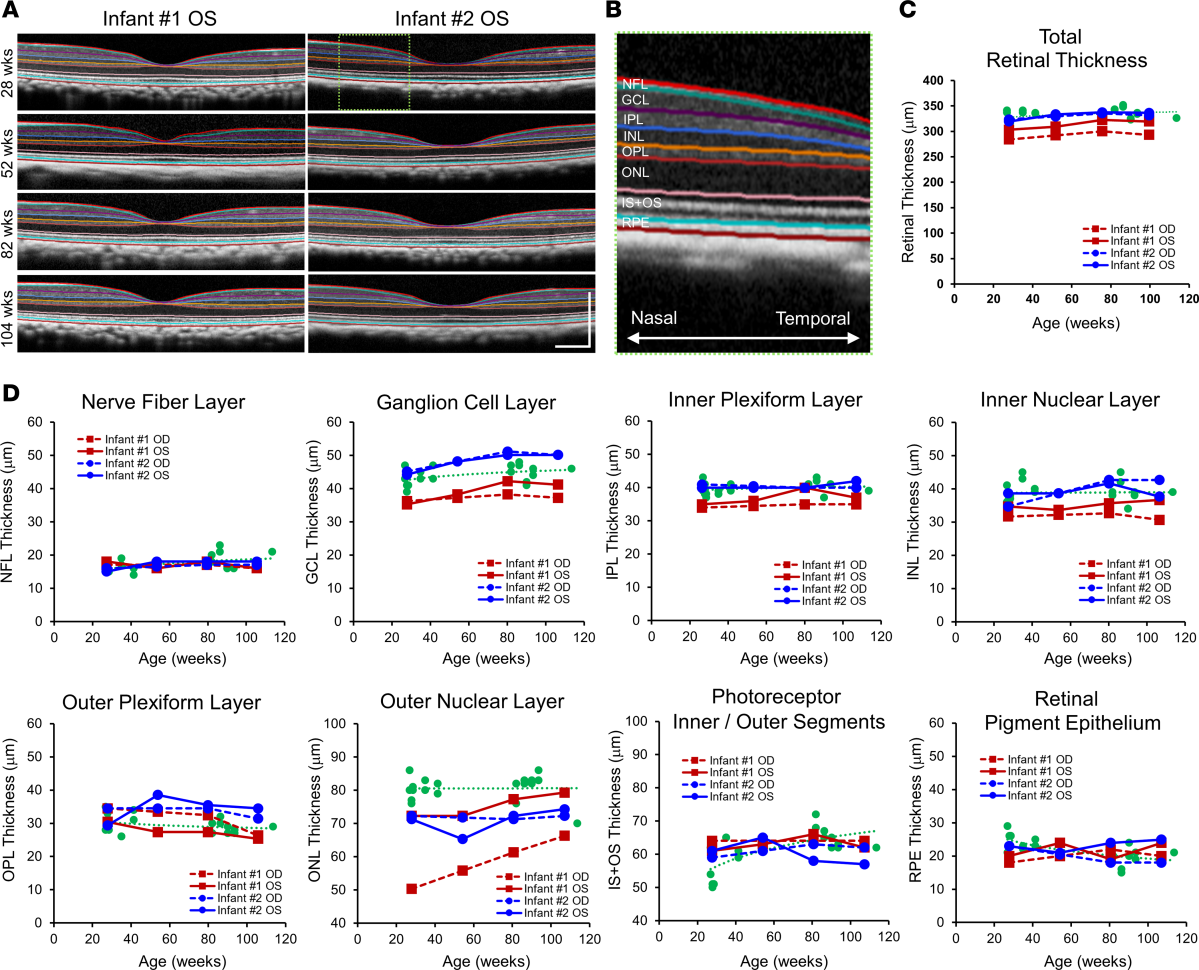
Retinal thinning in ZIKV-infected infant macaques. (**A**) Representative SD-OCT images of 2 infant macaques (no. 1 and no. 2) exposed to ZIKV infection in utero, with semiautomated segmentation of retinal layers. (**B**) Magnified view of the green dashed region of the SD-OCT image in **A** showing the retinal layers of the nasal parafoveal area, including the NFL, GCL, IPL, INL, OPL, ONL, IS+OS, and RPE. (**C**) Total retinal thickness and (**D**) individual retinal layer thicknesses measured from right (OD, dashed lines) and left (OS, solid lines) eyes from ZIKV-infected infant no. 1 (red) and no. 2 (blue), compared with eyes from healthy animals (green) and their trendline across similar ages. ZIKV, Zika virus; SD-OCT, spectral domain–optical coherence tomography; NFL, nerve fiber layer; GCL, ganglion cell layer; IPL, inner plexiform layer; INL, inner nuclear layer; OPL, outer plexiform layer; ONL, outer nuclear layer; IS+OS, photoreceptor inner and outer segments; RPE, retinal pigment epithelium; OD, right eye; OS, left eye.

Semiautomated segmentation of SD-OCT images enables precise, longitudinal measurements of different chorioretinal layers in the macula of rhesus macaque eyes with near-histological resolution (Figure 5, A and B) (44). Total retinal thickness was decreased in both eyes of infant no. 1 across all time points compared with infant no. 2 and control eyes (1.2–2.0 SD thinner in the left eye and 3.3–3.6 SD thinner in the right eye) (Figure 5C). Examination of individual retinal layers showed that most of the retinal thinning was a result of reduction in the GCL and outer nuclear layer (ONL), which consists of the cell bodies of retinal ganglion neurons and photoreceptors, respectively (Figure 5D). The thinning of these retinal layers was more pronounced in the right eye of infant no. 1, which exhibited the large chorioretinal coloboma, peripapillary atrophy, and macular dragging, and less severe in the left eye, which exhibited smaller colobomas and no macular distortion (Figure 5D). The other ZIKV-infected infant no. 2 did not show noticeable thinning in most retinal layers, except for the ONL, which was slightly reduced compared with healthy control eyes (Figure 5D).

### Ocular histopathology in ZIKV-exposed infant macaques.

Both animals were euthanized at approximately 2 years of age. The histology findings within the brain noted in the previous fetal studies (31) (changes in ependymal lining) were not observed in these infants, although minimal mineralization was observed in infant no. 2.

**Figure 6:**
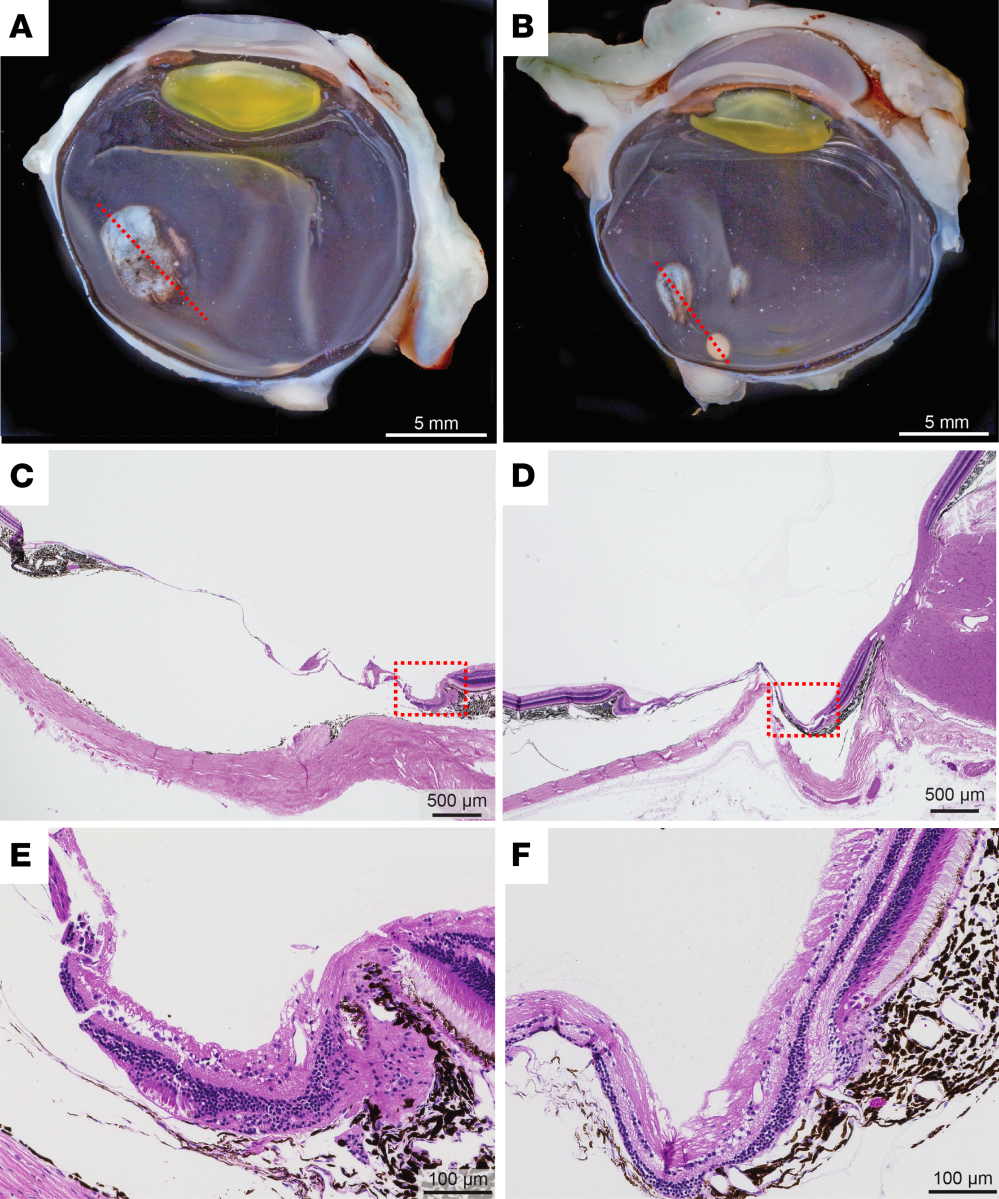
Gross pathology and histology of chorioretinal lesions in a ZIKV-infected infant macaque. (**A** and **B**) Macroscopic appearance of entire globes of ZIKV-infected infant no. 1 showing a single 5 mm oval, superotemporal chorioretinal defect in the right eye (**A**), and 2 chorioretinal defects of 2.5 mm and 1.5 mm in diameter nasal and superior to the optic nerve in the left eye (**B**). The red dashed lines show the orientation of histological sections. Scale bar: 5 mm. (**C** and **D**) H&E histological appearance of the chorioretinal lesions from the right (**C**) and left (**D**) eyes showing thinning of retina and choroid over sclera. Scale bar: 500 μm. (**E** and **F**) Magnified views of the chorioretinal lesion border near the red dashed boxed region in **C** and **D** show the transition from normal retina and choroid to the thin, dysplastic layers within the chorioretinal colobomas. Scale bar: 100 μm. ZIKV, Zika virus.

Macroscopic examination of the 2 eyes of infant no. 1 confirmed the presence of the chorioretinal colobomas (Figure 6, A and B), where histological analysis revealed disorganization and thinning of all retinal and choroidal layers. The retina in this area was reduced to a thin layer of dysplastic neuropil with scant glial cells, and the choroidal stroma was reduced to thin linear bundles of pigmented fibrous connective tissue that blend with the dysplastic retina (Figure 6, C–F). Neither eye of infant no. 2 demonstrated any pathologic histologic findings, including chorioretinal lesions or thinning of chorioretinal layers. Macroscopic pathology and histology of other major organ systems, including spleen, lymph nodes, lung, heart, jejunum, liver, kidney, spinal cord, and middle ear, did not reveal any lesions associated with ZIKV.

## Discussion

CZS is a devastating cause of congenital ocular malformations resulting from maternal ZIKV infection during pregnancy (8, 9, 11). However, the disease is poorly modeled in mice because rodents are not natural hosts of ZIKV and lack ocular anatomic features, such as the macula, which are unique to primate species. In this study, we employed a well-characterized model of CZS by infecting pregnant rhesus monkeys with ZIKV during the late first trimester and provided a detailed characterization of postnatal ocular development in 2 ZIKV-exposed infants over 2 years. We found that one of these animals exhibited large chorioretinal colobomas in both eyes, with macular dragging, peripapillary atrophy, and retinal thinning caused by loss of retinal ganglion neuron and photoreceptor layers that were more pronounced in the right eye of this animal. Despite the presence of these congenital ocular malformations, axial elongation and retinal development in ZIKV-infected infants appeared to follow normal postnatal maturation trajectories. The evolution of these ocular findings, along with the normal weight gain, absence of behavioral deficits, and loss of ZIKV-specific IgG after birth, suggests that active ZIKV infection and development of ocular defects occurred primarily in utero, with no indication of viral replication based on the absence of viral RNA in blood and CSF from infants and no continued impact on ocular development postnatally.

In our study, the ZIKV-exposed infant macaques exhibited congenital ocular anomalies in the absence of microcephaly or apparent neurological or behavioral deficits. This is similar to human CZS, in which ocular abnormalities have also been identified in patients with normal head circumference (10, 11), highlighting the need for eye screening among at-risk infants. In humans, the majority of ocular anomalies in CZS affect posterior segment structures, such as chorioretinal atrophy, torpedo maculopathy, and peripapillary atrophy (8, 9, 15, 18–20). These fundus findings are clinically descriptive but do not ascribe the pathologic cause of these types of lesions, which may occur as a result of trauma, infection, inflammation, or developmental defect, as in a chorioretinal coloboma. The pathogenesis of ocular abnormalities in CZS remains incompletely understood. Early studies identified ZIKV throughout the visual system, including the retina, optic chiasm, suprachiasmatic and lateral geniculate nuclei, and superior colliculus, which led to the hypothesis that ZIKV may be transmitted across the CNS through axonal transport (45). This is supported by SD-OCT retinal imaging of CZS infants that showed prominent thinning of the GCL, which consists of cell bodies of retinal ganglion neurons that send axon projections to the brain (21). However, additional studies also showed that ZIKV can effectively infect Muller glia as well as retinal vascular endothelium and RPE that respectively line the inner and outer blood–retinal barriers in mice, suggesting that circulating ZIKV can bypass these barriers to directly infect retinal tissues (28, 46). Our study supports this latter hypothesis based on (a) the presence of multiple ocular anomalies in the absence of neurological findings; (b) prominent thinning of ONL in addition to GCL, indicating loss of photoreceptors, which do not project directly to the CNS; and (c) constellation of macular dragging and peripapillary atrophy along the same meridian of the chorioretinal atrophy. These data suggest that these lesions are congenital colobomas potentially caused by infection of retinal progenitor cells during retinal development in utero rather than atrophic scars left by a ZIKV-related chorioretinitis. In fact, experimental models suggest that ZIKV does not directly infect photoreceptors (46, 47), and the ONL layers in the right eye of infant no. 1 increased with age, suggesting that the ONL thinning may result from mechanical distortion from the large coloboma rather than photoreceptor degeneration.

By characterizing the postnatal evolution of ocular abnormalities in ZIKV-exposed infants over 2 years, our study provides additional insight into the relationship between the ocular defects and in utero exposure to ZIKV detection in CZS. In previous studies that employed a similar model of combined i.v. and IA inoculation of pregnant macaques, when fetuses died in utero or were euthanized at the end of gestation or immediately after birth, the animals displayed diffuse viral tropism with the highest ZIKV RNA concentration found in neural, lymphoid, and cardiopulmonary systems, even though virus could not be found in cord blood plasma (31). In our study, the prolonged detection of viral RNA in amniotic fluid indicates the presence of viral replication in the fetal–placental compartment, which gradually declined toward the end of gestation, possibly due to increased transplacental transfer of maternal antibodies (48). Postnatally, the absence of viral RNA and gradual loss of antibodies in the infants support the lack of ongoing virus replication and thus insufficient antigen exposure to induce and sustain antibody responses. In our study of postnatal ocular development, despite the presence of congenital chorioretinal colobomas and retinal thinning in one infant, both axial length (Figure 2C) and total retinal layer thickness (Figure 5C) of both ZIKV-infected infants showed normal growth compared with healthy controls. Over the 2 years, the size of the chorioretinal colobomas remained unchanged (Figure 4A), and neither the retinal ganglion neurons in the GCL nor the photoreceptors in the ONL underwent further degeneration (Figure 5D). Thus, the in utero ZIKV infection appeared to be self-limited, and the ocular insult to the fetus occurred mostly during the early stages after infection. The absence of detectable viral RNA and loss of antibodies have been described in a human infant with ocular defects and CZS (19). This further highlights the difficulty of determining the long-term impact of CZS, because, for example, a child who presents to the clinic with neurological or ocular abnormalities but without a known history of ZIKV infection during pregnancy or detectable ZIKV or antibody, may pose difficulty establishing a causal relationship between the defect and in utero ZIKV exposure (49, 50).

To date, few studies have longitudinally followed the progression of congenital ocular anomalies in human infants with CZS. Using a well-established macaque model of CZS, our study showed that despite the presence of chorioretinal colobomas and retinal thinning at birth, postnatal ocular and retinal development appear to follow normal growth trajectory over the first 2 years of life, without evidence of active viral replication or further deterioration of ocular defects. Although we noted a slight anterior chamber shallowing and lens thickening in the animal with ocular pathology, we did not observe any visible anterior segment abnormalities, IOP elevation, or visual behavioral deficits. Long-term human studies in children with CZS could provide additional insight into the risk of glaucoma or cataracts in this pediatric population. Importantly, despite the stability of the ocular defects observed in this study, continued ophthalmic monitoring of suspected patients with CZS remains paramount to minimize the risks of amblyopia or long-term visual or neurological impairment.

## Methods

### Animals and care.

The adult female rhesus macaques (*Macaca mulatta*) in the study were born and raised in the conventional (not specific pathogen–free) breeding colony at the California National Primate Research Center (CNPRC). None of the animals were positive for type D retrovirus, SIV, or simian lymphocyte tropic virus type 1. All animals had prior successful pregnancies (range 2–6). For time-mated breeding, the female macaques were monitored for their reproductive cycles, and at the time of optimal receptiveness, they were temporarily housed with reproductively viable males. Pregnancy was confirmed via ultrasound. Gestational ages were determined from the menstrual cycle of the dam and the fetus length at initial ultrasound compared with growth data in the CNPRC rhesus macaque colony. Fetal health and viability were rechecked via ultrasound immediately before the first ZIKV inoculation and regularly thereafter. The 2 infants described in this report were born naturally by vaginal delivery on GD 168 (infant no. 1) or GD 171 (infant no. 2). Infants were reared by and lived with their mothers until they were approximately 17 months of age. At that time point the infants were then housed together until they were euthanized at 23–24 months of age. For control animals, ocular biometry and SD-OCT data from 10 age-matched rhesus macaques (mean age 62.5 ± 32.6 weeks, 6 males and 4 females) were randomly identified from the same colony and found to have no ocular abnormalities.

Macaques were housed indoor in stainless steel cages (Lab Product Inc.), the sizing of which was scaled to the size of each animal, as per national standards, and were exposed to a 12-hour light/dark cycle, 64°F–84°F, and 30%–70% room humidity. Animals had free access to water and received commercial chow (high-protein diet; Ralston Purina Co.), fresh produce 2 times per week, and forage (pea and oat mix) daily.

### Virus inoculations.

A combination of 2 virus isolates was used to inoculate the pregnant animals; these included a 2015 Puerto Rico isolate (PRVABC-59; GenBank, KU501215) and a 2015 Brazil isolate (strain Zika virus/H.sapiens-tc/BRA/2015/Brazil_SPH2015; GenBank, KU321639.1), which were used earlier in pregnant and nonpregnant animals (31, 40, 51). The use of 2 strains was intended to mimic a hyperendemic area where different variants may circulate. Aliquots of both virus stocks were kept frozen in liquid nitrogen, and new vials were thawed shortly before each inoculation. The inoculum was adjusted to 2000 PFUs (1000 PFUs of each strain) in 1 mL of RPMI-1640 medium, then kept on wet ice. Each pregnant animal was inoculated by both i.v. and IA routes, each route with 1 mL (2000 PFUs of the mixture). Whereas the normal gestation of rhesus macaques is 165 days, inoculations occurred between GDs 42 and 53, corresponding to the first trimester of human gestation. The 2 pregnant dams described in detail in this report were inoculated on estimated GD 51 (animal no. 1) or GD 53 (animal no. 2).

### Sample collection and clinical monitoring.

Macaques were evaluated twice daily for clinical signs of disease, including poor appetence, stool quality, dehydration, diarrhea, and inactivity. When necessary, macaques were immobilized with ketamine hydrochloride (Parke-Davis) at 10 mg/kg and injected intramuscularly after overnight fasting. Animals were sedated on days 0 (time of first virus inoculation; ~GD 30), 2, 3, 5, and 7 and then weekly for sample collection and ultrasound monitoring of fetal health. Fetal measurements were collected as previously described (40). After delivery, the animals were bled initially every few weeks, then less frequently. Blood was anticoagulated with EDTA and collected using venipuncture at every time point for complete blood counts (with differential count), and a separate aliquot of blood was centrifuged for 10 minutes at 800*g* to separate plasma from cells. The plasma was spun an additional 10 minutes at 800*g* to further remove cells, and aliquots were immediately frozen at –80°C.

Ultrasound-guided amniocentesis was conducted starting on day 7 after inoculation and then at all time points listed above according to methods described earlier (40). Amniotic fluid was spun to remove cellular debris, and the supernatant was aliquoted and immediately cryopreserved at –80°C for viral RNA assays.

### Isolation and quantitation of viral RNA from fluids and tissues for determination of infection status.

ZIKV RNA was isolated from samples and measured in triplicate by qRT-PCR according to methods previously described (40). According to the volume available, the limit of detection (LOD) for plasma and amniotic fluid ranged from 1 to 2.6 log_10_ viral RNA copies per mL fluid; because the average LOD was 1.4 log_10_ viral RNA copies, this was used as the LOD to graph Figure 1. For tissue, the limit of detection ranged from 3.2 to 3.5 log_10_ viral RNA copies/g tissue.

### Detection of ZIKV-specific binding IgG in macaque plasma.

ZIKV-specific binding IgG was detected using a whole virion ELISA previously described (48). Briefly, high-binding 96-well ELISA plates (Greiner) were coated with 40 ng/well of 4G2 antibody (clone D1-4G2-4-15) in carbonate buffer (pH 9.6 overnight at 4°C). Plates were blocked in Tris-buffered saline containing 0.05% Tween-20 and 5% normal goat serum for 1 hour at 37°C, followed by an incubation with ZIKV (PRVABC59 strain from BEI). Rhesus plasma was tested at 1:12.5 starting dilution in 8 serial 4-fold dilutions, incubating for 1 hour at 37°C. HRP-conjugated goat anti-human IgG monkey ads-HRP (Southern Biotech, 2049-05) was used at a 1:2500 dilution, followed by the addition of SureBlue reserve TMB substrate, followed by stop solution (KPL). Optical densities were detected at 450 nm (PerkinElmer, Victor). Half of maximal effective dilution (ED_50_) values were calculated with the sigmoidal dose-response (variable slope) curve fit in Prism 7 (GraphPad), which uses a least squares fit. The positive control was plasma from a ZIKV-infected monkey at 6 weeks after infection, and the negative control was plasma from an uninfected monkey. Samples with an ED_50_ below the limit of detection of 50 were plotted at the limit.

### Ophthalmic examination.

For detailed ophthalmic examinations, animals were sedated with ketamine hydrochloride, midazolam, and dexmedetomidine, followed by pupillary dilation with phenylephrine (Paragon Biotech), tropicamide (Bausch & Lomb), and cyclopentolate (Akorn). Ophthalmic evaluations were conducted by portable slit lamp biomicroscopy (SL-7E, Topcon) of the anterior segment and by indirect ophthalmoscopy (Heine) of the retinal fundus by a board-certified ophthalmologist and retinal specialist. IOP was measured by rebound tonometry (TonoVet, Icare). External photographs of the anterior segment were captured using a digital camera (Rebel T3, Canon). A-scan ultrasonography (Sonomed PacScan 300A+) was performed for measuring ocular biometry.

### Multimodal ocular imaging and analysis.

Color fundus photography was performed using the CF-1 Retinal Camera (Canon) with a 50-degree wide-angle lens. NIR, FAF, FA, and SD-OCT were performed using the Spectralis HRA+OCT system (Heidelberg Engineering), using a 30-degree or 55-degree objective for NIR, FAF, and FA imaging and the 30-degree objective for SD-OCT (52). Confocal scanning laser ophthalmoscopy was used to capture 30 × 30–degree NIR, FAF, and FA images using an excitation light of 820 nm for NIR and 488 nm for blue-peak FAF and FA imaging (53). For FA, animals were injected with 7.7 mg/kg fluorescein sodium (Akorn) by i.v. route, and serial images captured up to 15 minutes after dye injection. SD-OCT was performed using a 20 × 20–degree volume scan and a 30 × 5–degree raster scan protocol, centered on the fovea and in the areas of chorioretinal colobomas, with progression mode using retinal vessel tracking enabled, where possible, to reliably image the same area for longitudinal imaging sessions. All retinal measurements were made using the Heidelberg Explorer software (version 1.9.13.0, Heidelberg Engineering), which has been used in prior studies and calibrated for both humans (54–56) and macaques (57–59). Chorioretinal lesion diameters were measured from the widest horizontal dimension of each lesion on NIR imaging. Disc-to-fovea distance was measured from the visual center of the optic disc to the center of the foveal pit based on combined NIR and SD-OCT images. Semiautomated segmentation of the chorioretinal layers was performed by the Heidelberg Explorer software, followed by manual adjustment of the segmentation lines by a masked grader, including the nerve fiber layer, GCL, inner plexiform layer, inner nuclear layer, outer plexiform layer, ONL, photoreceptor inner and outer segments, and RPE. Average retinal layer thicknesses were measured from the nasal quadrant of the 1–3 mm ring of the Early Treatment of Diabetic Retinopathy Study grid (60) for consistency between animals.

### Necropsy and tissue collection for histopathology.

Animals were euthanized with an overdose of pentobarbital, followed by immediate collection of a specimen of spleen and inguinal lymph node (preserved in RNALater for RT-PCR) and upper body perfusion with 4% paraformaldehyde for optimal preservation of brains and eyes for histological analysis. Brains and eyes were collected immediately. The right hemisphere and both eyes were fixed further in 4% paraformaldehyde; the left hemisphere and other tissues were preserved in 10% neutral buffered formalin, routinely paraffin-embedded; and sections were stained with H&E and evaluated by board-certified anatomic pathologists. Histological sections were imaged using a ×40 objective lens on a Virtual Slide Microscope (VS120-S6-W, Olympus).

### Statistics.

Graphing and statistical analysis were performed with Prism 9 (GraphPad). *P* values of less than 0.05 were considered significant.

### Study approval.

Research was carried out at the CNPRC, which is accredited by the Association for Assessment and Accreditation of Laboratory Animal Care International. All studies using rhesus macaques (*Macaca mulatta*) followed the guidelines of the Association for Research in Vision and Ophthalmology Statement for the Use of Animals in Ophthalmic and Vision Research, complied with the *Guide for the Care and Use of Laboratory Animals* (National Academies Press, 2011), and were approved by the University of California, Davis, Institutional Animal Care and Use Committee.

## Author contributions

KKAVR conceived and designed the project. GY, MIC, AR, RIK, JW, JU, AS, EEB, EBM, WG, HW, and TS acquired the data. GY, AR, RIK, AS, EBM, WG, HW, TS, SP, AA, and LLC analyzed the data. GY and KKAVR drafted the manuscript, and all authors critically revised and edited the manuscript. GY, SMT, and KKAVR also provided administrative support.

## Version 1 11/12/2020

In-Press Preview

## Version 2 12/17/2020

Electronic publication
